# Molecular signature of response and potential pathways related to resistance to the HSP90 inhibitor, 17AAG, in breast cancer

**DOI:** 10.1186/1755-8794-3-44

**Published:** 2010-10-04

**Authors:** Magdalena Zajac, Gonzalo Gomez, Javier Benitez, Beatriz Martínez-Delgado

**Affiliations:** 1 Human Genetics Group, Spanish National Cancer Centre (CNIO), Madrid, Spain; 2 Bioinformatics Unit. CNIO, Madrid, Spain; 3 CIBERER, Centre for Biomedical Networking Research on Rare diseases, Madrid, Spain

## Abstract

**Background:**

HSP90 may be a favorable target for investigational therapy in breast cancer. In fact, the HSP90 inhibitor, 17AAG, currently has entered in phase II clinical trials as an anticancer agent in breast and other tumors. Since HSP90 inhibition leads to global depletion of oncogenic proteins involved in multiple pathways we applied global analysis using gene array technology to study new genes and pathways involved in the drug response in breast cancer.

**Methods:**

Gene expression profiling using Whole Human Genome Agilent array technology was applied to a total of six sensitive and two resistant breast cancer cell lines pre-treatment and treated with the 17AAG for 24 and 48 hours.

**Results:**

We have identified a common molecular signature of response to 17AAG composed of 35 genes which include novel pharmacodynamic markers of this drug. In addition, different patterns of HSP90 client transcriptional changes after 17AAG were identified associated to the sensitive cell lines, which could be useful to evaluate drug effectiveness. Finally, we have found differentially expressed pathways associated to resistance to 17AAG. We observed significant activation of NF-κB and MAPK pathways in resistant cells upon treatment, indicating that these pathways could be potentially targeted to overcome resistance.

**Conclusions:**

Our study shows that global mRNA expression analysis is a useful strategy to examine molecular effects of drugs, which allowed us the discovery of new biomarkers of 17AAG activity and provided more insights into the complex mechanism of 17AAG resistance.

## Background

Considering the complexity of breast cancer, with its multiple genetic abnormalities, targeting a single pathway by inhibiting the activity of one component is unlikely to be effective in a long term. Identification of molecular targets that will modulate multiple components of several signalling pathways would be desired for anticancer treatment. To that end, HSP90 gained lately extreme interest and became an interesting cancer drug target [1]. In breast cancer, preclinical studies have demonstrated sensitivity of HER2^+ ^tumors to HSP90 inhibitor [2-4], lately though it was demonstrated that HSP90 is a very effective target of therapy in triple negative breast cancers [5,6]. HSP90 is a chaperone for several oncogenic client proteins (ERBB2, B-RAF, CDK4, AKT, mutant p53, among others) involved in transcriptional regulation, signal transduction, and cell cycle control as well as in other crucial steps leading to malignant phenotype [7,8]. Hsp90 is overexpressed in tumor cells, indicating that these cells are highly dependent on the Hsp90 function [9]. Mutant oncoproteins may depend on the full function of Hsp90 as a conformational buffer to maintain full activity [10-13].

The HSP90 inhibitor, 17-allyloamino-17-demethoxy-geldanamycin (17AAG) a geldanamycin analogue, is currently in phase II clinical trials [14] in a number of cancers [15-19], see http://www.clinicaltrials.gov. At present, most of the drug candidates fail relatively late during the process (phase III) of clinical trials due to lack of efficacy [20,21]. To save that failure there is a high demand for biomarkers that can adequately and with great specificity, indicate the presence or absence of the desired pharmacological response[22]. Since HSP90 inhibition leads to global depletion of oncogenic proteins involved in multiple signaling pathways, expression signatures have been developed to understand the mechanisms of drug action and to predict the sensitivity to treatment. With the microarray technology, rather than studying effect of the drug on a single gene or protein, we can now look for signatures consisting of multiple genes that are altered in some way, and together define novel set of pharmacodynamic biomarkers of the drug response as well as of resistance[23]. Gene expression and proteomic profiling studies have been done previously in a panel of ovarian and colon cancer cell lines after 17AAG treatment [24,25] and in pancreatic cancer after treatment with 17AAG partner, IPI 504 [26], however there are no previous studies focused in breast tumors under 17AAG treatment.

Although a basic molecular signature of response to 17AAG has been previously defined [27] with depletion of the levels of client proteins such as c-RAF-1 and cyclin-dependant kinase 4 (CDK4), and upregulation of the inducible isoform of HSP70 (HSP72), the exact mechanism of action of 17AAG has not been clearly defined. To that end a discovery of clinical markers of response and mechanism of resistance to 17AAG are still a matter of time. The need to reveal biomarkers and understand the resistance will help to identify responsive versus non responsive patients to 17AAG. In our current study we performed a global gene expression analysis using Whole Human Genome array technology to understand the molecular mechanism of action of 17AAG in breast cancer. First, we have identified a breast cancer signature of response to 17AAG and suggested biomarkers of 17AAG sensitivity in breast cancer. Secondly, we have studied transcriptional changes in known HSP90 clients. And finally, we have identified gene expression and pathway activity differences in response to 17AAG in sensitive versus resistant cell lines.

All together these results may provide further understanding of the mode of action of 17AAG and suggest potential molecular markers of response and drug resistance in breast cancer.

## Methods

### Breast Cancer cell lines

Eight breast cancer cell lines were included in this study, MCF-7, MDA-MB-157, Hs578T, HCC1937, MDA-MB-436, UACC3199, MDA-MB-231 and T47D). Breast cancer cell lines, MCF-7, UACC3199, Hs578T, MDA-MB-231 and T47 D were obtained from Cancer Epigenetic Group at Spanish National Cancer Centre, Madrid, Spain. HCC1937 and MDA-MB-157 were kindly provided by Dr. P. Edwards from Department of Pathology, University of Cambridge, Cambridge, UK and MDA-MB-436 by Dr. K.S Massey-Brown from Department of Pharmacology and Toxicology, University of Arizona, Tucson, USA. These tumor cell lines were cultured in RPMI 1640 containing 10% fetal bovine serum (Gibco-BRL, Grand Island, NY, USA) with the exception of MDA-MB-157 being cultured in DMEM/F12 (Gibco-BRL) supplemented with 10% fetal bovine serum and maintained at 37°C in 5% CO_2. _All media were supplemented with fungizone, and penicillin/streptomycin.

Fresh material from a tumor biopsy corresponding to a breast tumor sample was used to validate results obtained in cell lines. Appropriate ethical committee approval and informed consent was obtained. Tumor was cultured *in vitro*, and after several passages, tumor cells were treated with 17AAG at the same dose used for the cell lines. These primary tumor cells were grown in F-10 medium (Gibco), supplemented with 10% fetal bovine serum and 2% Ultroser G (PALL Life Sciences).

### Drug and treatment protocol

17AAG (Sigma-Aldrich, St.Louis, MO, USA) was prepared as a 1 mM stock in dimethyl sulfoxide (DMSO) and stored at -20°C and freshly dissolved immediately prior to use. Cells were seeded in 10 cm dishes at a moderate density in 20 ml complete medium. At 24 h after plating, cells were treated with 500 nM 17AAG or DMSO (0.1%) as a control. At appropriate intervals 24 H, 48 H upon treatment with the drug, cells were harvested.

### RNA extraction, cRNA amplification, labeling and hybridization

Total RNA was extracted with TriReagent (Molecular Research Center, Cincinnati, OH, USA). Purity and integrity of the RNA was assessed with Agilent 2100 BioAnalyzer (Agilent Technologies, Palo Alto, CA, USA). Then, 500 ng of total RNA from samples and Universal Human Reference RNA (Stratagene, La Jolla, CA, USA) were used for amplification and labeling using the Agilent's Low RNA Input Linear Amplification Kit (Agilent Technologies) following the detailed protocol described in the kit manual. Cyanine 5 labeled samples or Cyanine 3 labeled reference cRNAs were purified using QIAGEN's RNeasy mini spin columns and eluted in 30 μl of nuclease-free water. After amplification and labeling, cRNA quantity and cyanine incorporation were determined using a nanodrop ND.1000 UV-VIS-Spectrophotometer version 3.2.1 (Agilent Technologies). For each hybridization,1 μg Cyanine 3 labeled cRNA (reference) and 1 μg of Cyanine 5 labeled cRNA (samples) were mixed, fragmented, and hybridized at 65°C for 17 hours to an Agilent 4 × 44 K Whole Human genome Oligo Microarray containing 45,015 features representing 41,000 unique probes. We have hybridized each sample at each time point (untreated, treated 24 H and 48 H with 17AAG), including some technical replicates. After washing, microarrays were scanned using an Agilent Array scanner (Agilent Technologies). Images were analyzed. Reproducibility and reliability of each single microarray was assessed using Quality Control report data. Data were extracted with Agilent feature extraction software (version 9.5.3) using the GE2-v5_95_Feb07 protocol. Background substraction were carried out using normexp. Lowess and quantiles methods were performed for intra-array and inter-array normalization respectively. Expression ratios were calculated (Cy5 processed signal was divided by Cy3 processed signal) and log2 transformed. Gene patterns containing missing values were discarded. Additionally a filter procedure eliminated genes with uniformly low expression or with low expression variation across the experiments, retaining 20374 genes and transcripts. Microarray dataset is publicly available at GEO database http://www.ncbi.nlm.nih.gov/geo/info/linking.html (GEO accession number, GSE23209).

### Differential gene expression analysis

To determine if there were genes differentially expressed between treated and untreated and sensitive versus resistant breast cancer cell lines supervised classification was performed with linear models (limma) implemented in the POMELO II tool, available in Asterias web server http://asterias.bioinfo.cnio.es. The estimated significance level (p value) was corrected for multiple hypotheses testing using Benjamini & Hochberg [28] False Discovery Rate (FDR) adjustment. Those genes with FDR <0.05 were selected as significantly differentially expressed.

### Clustering

Average linkage hierarchical clustering (Pearson correlation, uncentered metrics) from Gene Cluster and Treeview http://rana.stanford.edu/software algorithms were used to obtain clustering of the data sets. The level of expression of each gene in each sample, relative to the median level of expression of that gene across all the samples is represented using a red-white-blue color scale. Blue correspond to expression value below median, white, equal to median and red above the median.

### Functional Profiling of genes and pathways

We used Gene Set Enrichment Analysis (GSEA) to gain insight into global molecular networks and canonical pathways related to differentially expressed genes associated to resistant phenotype after 17AAG treatment. In our data analysis we have included the Biocarta http://www.biocarta.com as a source of pathway annotation, and whichgenes web-based tool for building 249 gene sets with application in gene set enrichment analysis [29]. The ranking of genes was performed with T test, with an absolute mode for gene list sorting. Gene set permutations were used to assess the statistical significance of the pathways. Those pathways showing FDR <0.05, a well-established cut-off for the identification of biologically relevant gene sets [30], were considered significantly enriched between classes under comparison. The ranking of genes was performed with T test, with an absolute mode for gene list sorting.

### Validation of microarray data by quantitative RT-PCR analysis

One μg of total RNA was reverse transcribed using MMLV Reverse Transcriptase (Invitrogen, San Diego, CA, USA) and random primers. The cDNAs were subjected to quantitative real-time PCR (QT-PCR) assay with the use of labeled probes for selected genes (Roche Universal Probe library, Basel, Switzerland) and the TaqMan Universal PCR Mix in an ABI prism 7900 system (Applied Biosystems Inc, Foster City, CA, USA) under manufacturer's recommendations. The PCR amplification was carried out with 10 min at 95°C, followed by 50 cycles of 15 s at 95°C and 1 min at 60°C, using the oligonucleotides shown in Additional file 1: Supplemental Table S1. All experiments were analyzed in triplicate.

### Validation of microarray data by western blot analysis

The protein expression changes were examined after 24 H and 48 H of 17AAG treatment. Western blot analysis was performed using standard procedures for whole-cell extracts from cell lines as described previously [31]. A set of antibodies used for immunodetection are listed in the Additional file 1: Supplemental Table S2. The immunoblotting was repeated at least twice in all of the experiments presented in this study.

### Validation of microarray data by cell based multi-pathway activity assays

Cignal Reporter Assay was performed according to manufacturer's instructions. Briefly, the transcription factor-responsive reporter, negative control, and positive control constructs were diluted in Opti-MEM (Invitrogen). The diluted nucleic acids were mixed with the diluted SureFECTTM (transfection reagent; SABiosciences) and delivered to 20,000 cells in a 96-well plate format. Culture media were changed 24 H after transfection. Transfection efficiency was estimated by following the expression of GFP (in the positive control wells) using fluorescence microscopy. At 24 H post transfection the cells were treated with the drug. After 48 H of treatment, cells were harvested into cell lysis buffer (Promega). Luciferase activities were determined using the Dual-Luciferase Assay System (Promega) and a multilabel reader (Promega). The Firefly/Renilla activity ratio generated from the transcription factor-responsive reporter transfections was divided by the Firefly/Renilla activity ratio generated from the negative control transfections to obtain the relative luciferase units. At least three independent transfections were carried out in triplicate for each of the conditions tested with each reporter assay. This experimental design was repeated at least once.

### Statistical analyses

Analyses of pathway activity differences upon treatment in the resistant and sensitive cell lines were performed using SPSS version 17.0 (SPSS Inc, Chicago, IL). Non-parametric, Mann-Whitney *U *test was used for our statistical hypothesis testing. P-values reported are two-sided.

## Results

### Identification of a molecular signature of response to 17AAG

Previous studies have shown feasibility of differential gene expression approach to create expression-based signatures predicting response or resistance in human tumors to therapeutic agents. Here, we investigated the biology underlying the differential gene expression across breast cancer cell lines treated with HSP90 inhibitor, 17AAG.

A total of eight breast cancer cell lines were analyzed. In a previous study, six of them (MCF-7, MDA-MB-157, Hs578T, HCC1937, MDA-MB-436, and UACC3199) were demonstrated to be sensitive to treatment with 17AAG in a dose and time dependent manner with IC50 values between 0.014 ± 0.006 μM and 0.059 ± 0.017 μM at 96 h [31]. In addition, two other cell lines were also analyzed (MDA-MB-231, T47D) and found to be resistant to 17AAG, showing IC50 values of 1.92 ± 0.4 μM and 3.82 ± 0.97 μM, respectively. Then, all cell lines were treated with 500 nM 17AAG for 24 and 48 hours, concentration at which sensitive cells showed cytotoxic and cytostatic effects as well as G2/M arrest and degradation of client proteins [31], and whole genome expression profiling of control and treated cells at this time points were performed.

**Figure 1:**
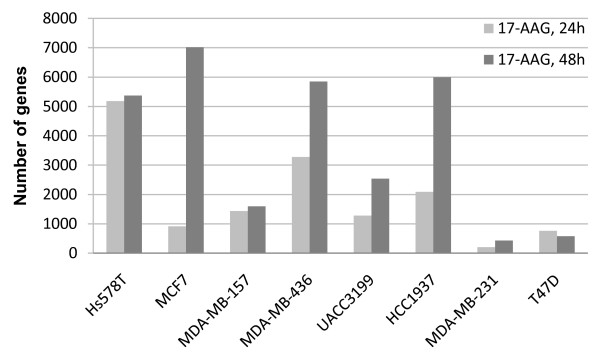
**Gene expression changes induced by 17AAG**. Averaged number of genes with expression changes >2-fold following 17AAG treatment are represented for all breast cancer cell lines, at both time points 24 H and 48 H. Resistant cell lines MDA-MB-231 and T47 D exhibited much lower variation after treatment.

Unsupervised hierarchical clustering of all samples using more than 20000 genes, as expected, did not separate the treated versus untreated cell lines. Instead, all 17AAG treated cell populations clustered with its control cell line, suggesting that the effect of the drug is very subtle in the whole genome context or that the effect of the drug is largely specific for each cell line (Additional file 1: Supplemental Figure S1). In general, after treatment with 17AAG, there was an increase in the number of genes with expression changes >2-fold over control cells after 48 h than after 24 h. Roughly, the proportion of genes with >2-fold changes at 48 h ranged between 8% and 35% in sensitive cells. Resistant cell lines showed lower number of genes, only 2% (MBA-MD-231) and 2.8% (T47D) of genes showing >2-fold change versus control (Figure 1). Significantly, only a small number of genes were commonly altered in the resistant cell lines after treatment.

**Figure 2:**
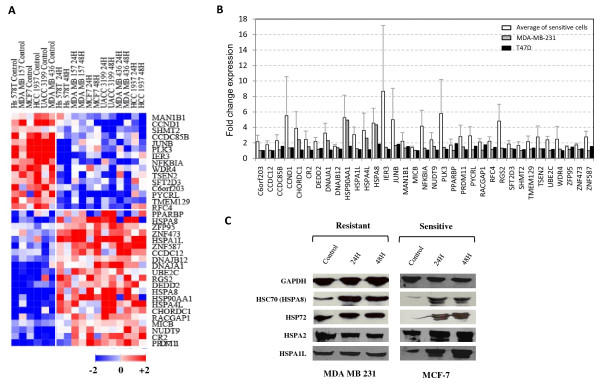
**Gene signature of response to 17AAG in breast cancer cell lines**. A) Heat-map represents the expression of significant differentially expressed 35 genes included in the molecular signature of response to 17AAG. Untreated samples were compared to treated samples from all sensitive cell lines to obtain common variations. B) Comparison between the changes in expression in the genes included in the signature in sensitive and resistant cells. Fold changes after 17AAG for sensitive cell lines, taken the averaged expression of all of them, compared to fold changes in resistant MDA-MB-231 cells or T47 D.Error bars represent standard deviation. C) Western blot analysis of different HSP70 isoforms before and after 17AAG in resistant MDA-MB-231 and sensitive MCF-7 cell line showed differential HSP70 induction after 17AAG. Lack of induction of the HSPA2 and HSPA1L in resistant cells, and increased expression of HSP72 and HSC70 in both resistant and sensitive cell lines upon treatment is shown. GAPDH was used as loading control.

Regardless of the specific changes found in each cell line, we searched for genes commonly associated to sensitivity to 17AAG. Analysis of differentially expressed genes between all treated compared with all untreated 17AAG sensitive breast cancer cell lines was performed. A list of 35 genes with a significant FDR value <0.05 was obtained, which constituted a molecular signature of response to 17AAG in breast cancer cells (Figure 2A). Among the 35 genes, 15 were down-regulated and 20 up-regulated genes following treatment (Table 1). The up-regulated genes following treatment included heat shock family proteins such as *HSP90AA1, HSPA8, DNAJB12, HSPA1L*, *DNAJA1, HSPA4L*, consistently with other array studies in different tumor cell types [24,32,33]. Among other genes showing increased expression after treatment were ubiquitin-conjugating enzyme E2C (*UBE2C*), death effector domain containing 2 (*DEDD2*), zinc finger protein 587 (*ZNF587*), zinc finger protein 473 (*ZNF473*), Rac GTPase activating protein 1 (*RACGAP1*), MHC class I polypeptide-related sequence B (*MICB*), regulator of G-protein signalling 2 (*RGS2*), cysteine and histidine-rich domain (CHORD)-containing 1 (*CHORDC1*), PPAR binding protein. There were also down-regulated genes in response to 17AAG which included e.g. *JUNB*, *CYCLIN D1*, *NFKBIA*, immediate early response 3 (*IER3*), transmembrane protein 129 (*TMEM129*) or replication factor C (activator 1) 4 (*RFC4*).

**Table 1:** Molecular signature of 17AAG response in breast cancer

20 Genes up-regulated following 17AAG treatment			
Gene name	Description	FDR	Fold Change
CHORDC1	Cysteine and histidine-rich domain (CHORD)-containing 1 (CHORDC1)	0.011	3.9

DEDD2	Death effector domain containing 2 (DEDD2)	0.011	2.2

HSPA8	Heat shock 70kDa protein 8 (HSPA8) transcript variant 1	0.011	4.6

NUDT9	Nudix (nucleoside diphosphate linked moiety X)-type motif 9 (NUDT9). transcript variant 1	0.011	2.4

PRDM11	PR domain containing 11 (PRDM11)	0.011	2.8

UBE2C	Ubiquitin-conjugating enzyme E2C (UBE2C)	0.011	2.4

ZFP95	Zinc finger protein 95 homolog (mouse) (ZFP95). transcript variant 1	0.02	1.5

ZNF587	Zinc finger protein 587 (ZNF587) transcript variant 1	0.025	2.8

RGS2	Regulator of G-protein signalling 2. 24kDa (RGS2)	0.03	4.8

CR2	CR2/CD21/C3d/Epstein-Barr virus receptor mRNA. complete cds. [M26004]	0.034	2.5

DNAJA1	DnaJ (Hsp40) homolog. subfamily A. member 1 (DNAJA1)	0.034	3.3

HSPA1L	Heat shock 70kDa protein 1-like (HSPA1L)	0.034	3.1

MICB	MHC class I polypeptide-related sequence B (MICB)	0.034	1.4

RACGAP1	Rac GTPase activating protein 1 (RACGAP1)	0.034	2.1

ZNF473	Zinc finger protein 473 (ZNF473). transcript variant 1	0.034	1.7

DNAJB12	DnaJ (Hsp40) homolog. subfamily B. member 12 (DNAJB12). transcript variant 1	0.035	1.6

HSP90AA1	Heat shock protein 90kDa alpha (cytosolic). class A member 1 (HSP90AA1) transcript variant 2	0.045	5.3

PPARBP	PPAR binding protein. mRNA (cDNA clone IMAGE:4822636). complete cds. [BC060758]	0.045	1.7

CCDC12	Coiled-coil domain containing 12 (CCDC12)	0.046	1.8

HSPA4L	Heat shock 70kDa protein 4-like (HSPA4L)	0.046	3.6

**15 Genes down-regulated following 17AAG treatment**			
**Gene name**	**Description**	**FDR**	**Fold Change**

PLK3	Polo-like kinase 3 (Drosophila) (PLK3)	0.025	5.8

TMEM129	Transmembrane protein 129 (TMEM129)	0.027	2.2

IER3	Immediate early response 3 (IER3). transcript variant short	0.032	8.7

CCND1	Cyclin D1 (CCND1)	0.034	5.5

JUNB	Jun B proto-oncogene (JUNB)	0.034	5

TSEN2	tRNA splicing endonuclease 2 homolog (S. cerevisiae) (TSEN2)	0.034	2.8

CCDC85B	Coiled-coil domain containing 85B (CCDC85B)	0.035	2.3

WDR4	WD repeat domain 4 (WDR4). transcript variant 2	0.035	2.5

RFC4	Replication factor C (activator 1) 4. 37kDa (RFC4). transcript variant 1	0.044	2.2

PYCRL	Pyrroline-5-carboxylate reductase-like (PYCRL)	0.045	2.9

SHMT2	Serine hydroxymethyltransferase 2 (mitochondrial) (SHMT2)	0.045	1.7

C6orf203	Chromosome 6 open reading frame 203 (C6orf203)	0.046	2.2

MAN1B1	Mannosidase. alpha. class 1B. member 1 (MAN1B1)	0.046	2.2

NFKBIA	Nuclear factor of kappa light polypeptide gene enhancer in B-cells inhibitor. alpha (NFKBIA)	0.047	4.2

SFT2D3	SFT2 domain containing 3 (SFT2D3)	0.047	1.9

As expected, most of the 35 genes from the signature of response did not change upon treatment in the resistant cell lines. However, some members of the co-chaperone complex such as, *HSP90AA1, HSPA4L, HSPA8, DNAJA1 *and *CHORDC1 *showed induction following treatment, in the resistant MDA-MB-231 cell line, but not in T47 D cells (Figure 2B). Interestingly, HSP90 and HSP70 induction are common markers of response to 17AAG. Indeed, HSP70 is one of the most commonly used pharmacodynamic markers of HSP90 inhibition in clinical trials nowadays. However, we have seen induction of the most common isoforms of HSP70, HSPA8 (HSC70) and HSP72, not only in sensitive cell lines but also in MDA-MB-231 resistant cell line. The induction of *HSP90, HSPA8 (HSC70) *and *HSP72 *in MDA-MB-231 cells was confirmed by QT-PCR and western blot (Figure 2C). We also analyzed by western blot other isoforms of HSP70, such as HSPA1L, which was part of the signature of response, and HSPA2 (Figure 2C). These two isoforms, in contrary to HSC70 and HSP72 were found exclusively induced in responsive cell lines upon treatment, suggesting that they could be better markers of sensitivity to 17AAG. Moreover, down or up-regulation of other differentially expressed genes from the molecular signature of response to 17AAG was also validated by QT-PCR (Additional file 1: Supplemental Figure S2).

### Validation of the signature of response in a tumor biopsy

**Figure 3:**
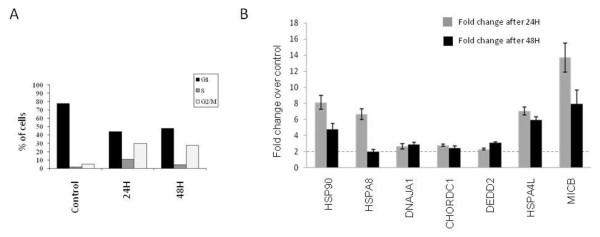
**Effect of HSP90 inhibition by 17AAG in a biopsy from a breast cancer patient**. A) Cell cycle analysis in untreated cells and cells treated with 17AAG for 24 H and 48 H, exhibiting G2/M arrest at both time points. B) Validation of a group of genes from the signature of response to 17AAG in the primary tumor cells. All these genes showed increased expression after treatment >2fold at 24 H or 48 H, as occurred in the sensitive cell lines.

To validate and investigate the significance of the obtained molecular signature of 17AAG response, a breast tumor biopsy from a patient with breast cancer was grown *in vitro *and treated with the same concentration used for tumor cell lines for 24 h and 48 h. Similarly to what was seen in cell lines [31], 17AAG produced in these primary tumor cells a growth inhibitory effect, with an arrest in G2/M phase of the cell cycle (Figure 3). Then, changes in the expression of a group of seven genes from the signature (*HSP90, HSPA8, DNAJA1, CHORDC1, DEDD2, HSPA4L *and *MICB*) were assessed by QT-PCR comparing with untreated cells. Significantly all these genes were confirmed to be 17AAG-responsive genes, also in this primary tumor.

### Transcriptional changes in HSP90 client proteins

**Figure 4:**
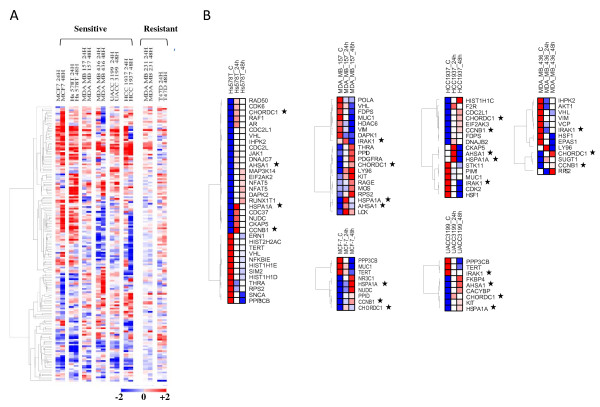
**Transcriptional changes of HSP90 clients**. A) Heatmap representing the differences in the mRNA expression found in the list of known HSP90 clients and interactors in treated breast cancer cell lines (increments over untreated cells in red, reductions in blue). Weaker variations in resistant cell lines are shown. B) Significant HSP90 client transcriptional changes in individual cell lines following 17AAG treatment. Genes for which mRNAs exhibited over 2-fold increase or reduction are shown for the different cell lines. Stars indicate genes commonly changing at least in four of the six cell lines.

The cellular response to 17AAG has a complex nature with effects including protein and transcriptional changes [25]. As the treatment with 17AAG has a global effect in the cell through depletion of client proteins, we were interested in analysing the subsequent changes induced by HSP90 inhibition at transcription level. We characterized the profile of transcriptional changes of the list of 168 well reported Hsp90 client proteins and interactors available on the Picard lab home page http://www.picard.ch/DP/DPhome.html. Since expression of HSP90 client proteins vary according to cell type, we have presented the data of HSP90 interactors' expression as differences in expression in treated cell lines versus their corresponding untreated cells. This analysis revealed that each cell line has an individual pattern of Hsp90 interactors' changes (Figure 4A). As we expected, variations in the expression levels of genes coding for client proteins were much lower in resistant cell lines comparing to the sensitive ones (Figure 4A), suggesting that transcriptional changes of at least some client proteins could be taken into account to measure drug effectiveness.

To identify crucial differentially expressed HSP90 clients in each cell line, expression changes of >2 fold after treatment were obtained. Indeed, the sensitive cell lines showed a much higher number of HSP90 clients that differ following treatment, compared to the resistant cell lines. By selecting HSP90 interactors that change >2 fold at both time points, 24 h and 48 h following 17AAG, Hs578T cells showed 35 genes, MCF-7 showed 9 genes; MDA-MB-157 showed 20 genes; MDA-MB-436 showed 13 genes; HCC1937 showed 17 genes and UACC3199 showed 9 genes (Figure 4B), while the resistant cell lines, T47 D showed only 3 genes and in MDA-MB-231 only 2 genes changed. Moreover, there were only two HSP90 interactors commonly upregulated in all the sensitive cell lines: *HSPA1A (HSP72)*, the inducible isoform of HSP70, and *CHORDC1*. Interestingly, the resistant cell line MDA-MB-231 showed exactly these two genes *HSPA1A *and *CHORDC1 *upregulated at both time points with no other HSP90 interactor significantly changing its expression. Upregulation of *HSPA1A *was detected also in T47 D resistant cells only after 48 h post treatment.

In addition to *HSPA1A *and *CHORDC1*, other client protein transcripts expression such as *ASHA1 *and *CCNB1 *were commonly induced, and *IRAK1 *decreased, with the treatment in at least four of the six cell lines (Figure 4B).

### Signaling Pathways related to resistance to 17AAG

It is well established that a major factor in resistance to 17-AAG is expression/activity of DT-diaphorase (NQO1), [34,35]. The resistant cell line, MDA-MB-231, has very low level of NQO1 [34], and it is likely to be the main cause of 17AAG resistance in this cells. In contrary, the other resistant cell line T47 D has higher levels of NQO1 and may have another, independent mode of resistance to 17AAG. Regardless of the intrinsic mode of resistance in the two cell lines, we investigate genes and pathways differentially modulated in responsive and resistant cells following treatment.

Differential expression profiling between resistant and sensitive cell lines after treatment was performed. As expected, large differences were found, with more than 1500 genes significantly changing (data not shown). Many of these differences could be attributed to the fact that sensitive treated cells represent arrested cells, while resistant cells are proliferating cells. In fact, significant number of these genes was involved in cellular metabolism.

Then, we tried to find whether there were significant signaling pathways that could be differentially expressed between treated sensitive and resistant cells. In order to obtain significant pathways, gene set enrichment analysis (GSEA) was performed. Because of the small number of samples this analysis did not show any pathways associated with 17AAG resistance with a FDR < 0.05. Even though the three top pathways more associated to 17AAG resistance included CDMAC pathway (FDR = 0.157), NF-κB pathway (FDR = 0.205) and ATM pathway (FDR = 0.246) (See Additional file 1: Supplemental Table S3, for a list of the top 10 pathways related to 17AAG resistant cells).

Additionally to the *in silico *pathway analysis, to look for differentially activated pathways following 17AAG treatment we have applied transcription factor-responsive luciferase reporter assays to resistant (T47 D, MDA-MB-231) and two sensitive cell lines (Hs578T, MCF-7). The latter quantitatively assesses the signal transduction pathway changes by measuring the activities of downstream transcription factors. By this method we evaluated activation of ten different cancer related pathways such as NOTCH, WNT, TGFβ, P53, cell cycle, MYC/MAX, NF-κB, MAPK/ERK, MAPK/JNK and HIF1.

**Figure 5:**
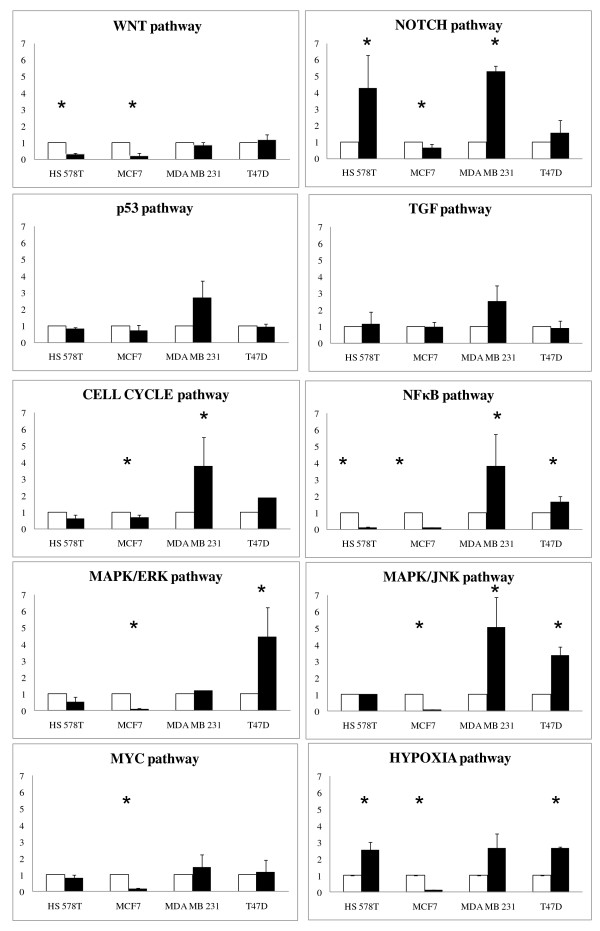
**Differential signaling pathway activation in resistant and sensitive breast cancer cells upon treatment with 17AAG**. Transfections were carried out in the sensitive (MCF-7 and Hs578T) and resistant (MDA MB 231 and T47D), cells with the reporter constructs for the typical cancer biology pathways: NOTCH, WNT, TGFβ, P53, cell cycle, MYC/MAX, NF-κB, MAPK/ERK, MAPK/JNK and HIF, followed by 17AAG or DMSO treatment. Dual-luciferase assay was performed, and promoter activity values are expressed as arbitrary units using a Renilla reporter for internal normalization. The experiment performed itself in triplicates was repeated at least twice of each cell line studied. The error bars represent standard deviation. Pathways significantly (p-value < 0.05) repressed or activated are marked with a star.

Interestingly, the results revealed that NF-κB and MAPK/JNK pathway were found significantly activated in both resistant compared to the sensitive cell lines (Figure 5). Although below statistical significance, GSEA revealed NF-κB as one of the most relevant pathways associated to 17AAG resistance. The increased activity of the NF-κB, may indicate that this pathway plays an important role in cell survival upon treatment. Additionally, MAPK pathways could have also some role in the resistance to 17AAG, as MAPK/JNK was significantly activated in both resistant T47 D and MDA-MB-231 cell lines, and the MAPK/ERK was induced in T47 D. Cell cycle pathway was significantly activated only in MDA-MB-231 resistant cell line, what may reflect the higher proliferation status of these cells. Sensitive MCF-7 cells showed a critical reduction in the activity of all of the pathways, which correlate with the stop in proliferation and death induced after the treatment (Figure 5). The other sensitive cell line, Hs578T also showed inhibition of most of the pathways except NOTCH, TGF and Hypoxia pathway.

## Discussion

In an attempt to identify detailed molecular mechanisms of drug response and resistance to treatment with 17AAG we performed gene expression profiling of eight breast cancer cell lines treated with the drug. Although there are many studies analyzing the molecular effect of HSP90 inhibition, global expression changes after 17AAG in breast cancer have not been analyzed in-depth. In this study we examined the expression changes in the sensitive (MCF-7, MDA-MB-157, Hs578T, UACC3199, HCC1937, MDA-MB-436) and two resistant (MDA-MB-231, T47D) breast cancer cell lines to the HSP90 inhibitor, 17AAG.

Gene expression profiling after 17AAG, showed different number of 17AAG responsive genes associated to the different cell lines. As HSP90 inhibition results in degradation of client proteins, it is possible that intrinsic differences in the abundance of HSP90 clients in the cells cause subsequent transcriptional changes in a cell line dependent manner. Although it was clear that HSP90 inhibition produced cell line dependent changes we could not associate them to the fact that these cell lines belong to different molecular breast cancer subtypes. Since one of the resistant cell lines, MDA-MB-231, is reported to be basal B and the other, T47 D, as luminal, and the rest of the sensitive cell lines being all basal B except for MCF7 (luminal) and HCC 1937 (basal A) [36] it is likely that sensitivity to 17AAG is not connected to known breast cancer subtypes.

However, there were a group of genes commonly regulated in all sensitive cell lines upon treatment. We have identified a breast cancer associated molecular signature of response to 17AAG, consisting of 35 17AAG-responsive genes. This gene signature of 17AAG response included, similar as previously reported in other studies [24-26], members of the chaperon complex, *HSP90 *itself and *HSP70 (HSPA8)*. These changes were identified in a study done in an ovarian cancer cell line as likely on-target effects of the drug, which are induced as a direct consequence of HSP90 inhibition [25]. Importantly, HSP70 isoforms have been used as pharmacodynamic end point in clinical trials [27]. In addition to these chaperons, we also identified up-regulation of other members of heat shock response family such as *HSPA4L, HSPA1L, HSP40 (DNAJA1 *and *DNAJB12) *and in *CHORDC1*, another HSP90 binding protein. Interestingly, we found transcriptional induction of *HSP90, HSP70 (HSPA8) *and *HSPA4L *in one of the resistant cell lines analyzed, MDA-MB-231. Moreover, HSC70/HSPA8 and HSP72 induction was confirmed by western blot in these cells. The up-regulation after treatment of HSP90 and HSP70 (both HSC70 and HSP72) might suggest that HSP90 is inhibited at 500 nM 17AAG in both sensitive and MDA-MB-231 resistant cell line. Yet, the resistance in the MDA-MB-231cell lines might be caused by very low levels of NQO1, as reported previously [37]. HSC70 and HSP72 also have an antiapoptotic role [38], so their induction in MDA-MB-231 might also contribute to the 17AAG resistance in these cells. The other resistant T47 D cells seem not to show the induction of Hsp70 isoforms following treatment what could suggest lack of HSP90 target inhibition by 17AAG. Interestingly, T47 D cells have some expression of NQO1 and probably an alternative mechanism of resistance.

The analysis of a number of HSP70 isoforms by immunoblotting revealed, in addition to induction of HSC70 and HSP72, that some other HSP70 isoforms were also induced by 17AAG. Up-regulation of HSPA1L and HSPA2 were found in sensitive cells following exposure to 17AAG. However, they showed lack of induction in resistant MDA-MB-231 cells. We suggest that HSPA1L and HSPA2 could represent potential biomarkers to follow up the effectiveness of 17AAG in breast cancer, although the mechanism underlying this effect is still unclear.

Other genes from the signature also exhibiting increased expression in response to 17AAG were Rac GTP-ase activating protein (*RACGAP1*), ubiquitin conjugating enzyme E2C (*UBE2C*), zinc fingers proteins (*ZNF473, ZNF587*) and MHC class I antigen (*MICB*). These data are in line with previous work done on 17AAG treated ovarian cancer cell lines [25]. Additionally, in a recent microarray study of novel HSP90 inhibitor (IPI-504) in pancreatic cancer, Song and colleagues [26] identified similar class of up-regulated genes following treatment along with GTPase activating proteins, zinc finger proteins, heat shock proteins and ribosomal proteins.

There were also genes with decreased expression following HSP90 inhibition by 17AAG. Some of them clearly represent cell cycle regulators (*CCND1, PLK3*) and important proliferation signaling pathways mediators (*JUNB, NFKBIA*). The decreased expression of them might be a consequence of cell cycle arrest produced after 17AAG.

The fact that the expression changes seen in the primary tumor sample after treatment with 17AAG resembled the changes in cell lines, suggests that this set of genes would constitute a robust signature of response in breast cancer. Further studies in additional tumor biopsies are required to better establish the value of the biomarkers identified in this study.

Since effects of 17AAG are driven by HSP90 client proteins degradation, we were interested in studying whether protein depletion also results in transcriptional changes of known client proteins following treatment. Changes in the mRNA levels of a number of client proteins were evident in cell lines responsive to 17AAG, while resistant cell lines demonstrated insignificant variations in transcriptional levels of HSP90 interactors. This observation suggests that the use of transcriptional changes of HSP90 client proteins may facilitate the selection of potentially responsive patients to 17AAG therapy. It is known that client proteins are variable in different types of tumor [10]. It is reasonable then, to find cell line specific transcriptional changes profiles in the 17AAG sensitive cell lines. This finding could be of interest in order to define, in further studies, key client proteins for specific tumor subtypes, with potential clinical significance. In addition, consistently up or down-regulated HSP90 client transcripts following treatment were identified shared by some of the cell lines analyzed (*AHSA1*, *CCNB1, IRAK1)*, that could represent important HSP90 clients in breast cancer. It is clear that biological processes are regulated not only at transcriptional level, but also protein levels or posttranscriptional modifications of proteins are important when analyzing the effects of HSP90 inhibitors. However, mRNA changes could be helpful in order to evaluate the effect of the drug in clinical samples.

Mechanisms of resistance to 17AAG remain largely unknown. We analyze global expression changes after 17AAG occurring in resistant cells, to define genes or pathways commonly involved in insensitivity to this drug. The identification of pathways in relation to 17AAG resistance would be important to develop in future candidate treatments to be used in combination with 17AAG to induce growth inhibitory effects in the insensitive cell lines. Functional studies with the pathway-focused reporter assays shown significant up-regulation of NF-κB pathway in resistant cells after exposure to 17AAG. It is interesting that GSEA also showed NF-κB pathway as potentially involved in resistance to 17AAG. In addition, there are two other resistance associated pathways in the top-ten list that were related to inflammation/immune response (NTHI_PATHWAY and INFLAM_PATHWAY) (Additional file 1: Supplemental Table S3). Thus it is possible that resistant breast cancer cell lines make use of the inflammation/immune response machinery to evade cell cycle arrest or apoptosis after 17AAG treatment. NF-κB activation was already observed after treatment with cancer chemotherapeutic agents such as gemcitabine [39-41], thereby inducing resistance to apoptosis which results in poor clinical outcome. NF-κB is a ubiquitously expressed transcription factor that is involved in a wide spectrum of cellular functions including cell cycle control, stress adaptation, inflammation and control of apoptosis [42]. Activation of NF-κB has been implicated in the development of a number of human malignancies, and it appears to be important for the survival of cancer cells, as well as the conferring of more aggressive tumor phenotype and resistance to drug therapies [43-46]. Moreover, high basal levels of NF-kB have been related with resistance to gemcitabine in pancreatic carcinoma cell lines [41,45]. The finding of NF-kB pathway activation after 17AAG treatment significantly associated to resistant cell lines suggests that simultaneous use of anti-tumor agents that block NF-κB activity together with HSP90 inhibitors may have a greater therapeutic value. MAPK/JNK, and also MAPK/ERK pathway were activated in the resistance cell lines, suggesting these pathways may be important for survival of cells after 17AAG treatment.

In summary, in this study we have established a 35 gene-based molecular signature of response to 17AAG in breast cancer, which revealed novel pharmacodynamic markers of drug response. Secondly, we have defined transcriptional changes in known HSP90 client proteins, which may be useful to monitor drug efficacy and finally, we have identified signaling pathways differentially activated following 17AAG in resistant cell lines.

## Conclusions

This study confirms gene expression profiling as a useful tool in further understand molecular response to drugs and in the discovery of novel pharmacodynamic markers. By analyzing gene expression changes induced by 17AAG in breast cancer cells we have identified a novel breast cancer associated molecular signature of response to 17AAG, consisting of 35 17AAG-responsive genes which might be potential biomarkers of sensitivity to evaluate clinical response. Analysis of mRNA changes of known HSP90 clients demonstrated cell line specific patterns associated with responsive cells, so this approach could be useful for the follow up of treatment response and could facilitate the discovery of key clients. In addition, signaling pathways associated with resistance to 17AAG were found, such as induction in DNA-binding activity of NF-κB in resistant cells after treatment. The identification of pathways associated to resistance would enable the design of combined therapies to overcome 17AAG resistance.

## List of abbreviations

**17AAG**: 17-allyloamino-17-demethoxy-geldanamycin; **DMSO**: Dimethyl sulfoxide; **FDR**: False Discovery Rate; **GSEA**: Gene Set Enrichment Analysis; **QT-PCR**: Quantitative real-time Polymerase Chain Reaction.

## Competing interests

The authors declare that they have no competing interests.

## Authors' contributions

MZ carried out hybridizations, participated in the analysis of results and have been involved in drafting the manuscript. GG gave support for the analysis and interpretation of microarray data and performed the statistical analysis. JB participated in the design of the study and analysis of results. BMD conception and design of the study, supervision of experiments and helped to draft the manuscript. All authors read and approved the final manuscript.

## Pre-publication history

The pre-publication history for this paper can be accessed here:

http://www.biomedcentral.com/1755-8794/3/44/prepub

## Supplementary Material

Additional file 1 **Supplemental tables and figures**. Supplemental Table S1: Oligonucleotides used for quantitative RT-PCR to validate expression of the selected from microarray study. Supplemental Table S2: List of the antibodies used for western blot. Supplemental Table S3: Top-ten pathways more associated to resistance to 17AAG. Supplemental Figure S1: Unsupervised clustering showed that samples mostly grouped by cell line. Some samples have technical replicates (marked with a star). Supplemental Figure S2: QT-PCR measurements of selected genes from the signature, confirmed the down regulation of five genes from the molecular signature (*CYCLIN D1, JUNB, TMEM129, PLK3, NFKBIA*) and up-regulation of *UBE2C *after treatment. Click here for file

## References

[B1] Eccles SA Massey A Raynaud FI Sharp SY Box G Valenti M Patterson L de Haven Brandon A Gowan S Boxall F NVP-AUY922: a novel heat shock protein 90 inhibitor active against xenograft tumor growth, angiogenesis, and metastasis Cancer Res 2008 68 8 2850 2860 10.1158/0008-5472.CAN-07-5256 18413753

[B2] Chiosis G Timaul MN Lucas B Munster PN Zheng FF Sepp-Lorenzino L Rosen N A small molecule designed to bind to the adenine nucleotide pocket of Hsp90 causes Her2 degradation and the growth arrest and differentiation of breast cancer cells Chem Biol 2001 8 3 289 299 10.1016/S1074-5521(01)00015-1 11306353

[B3] Neckers L Heat shock protein 90 inhibition by 17-allylamino-17- demethoxygeldanamycin: a novel therapeutic approach for treating hormone-refractory prostate cancer Clin Cancer Res 2002 8 5 962 966 12006507

[B4] Modi S Stopeck AT Gordon MS Mendelson D Solit DB Bagatell R Ma W Wheler J Rosen N Norton L Combination of trastuzumab and tanespimycin (17-AAG, KOS-953) is safe and active in trastuzumab-refractory HER-2 overexpressing breast cancer: a phase I dose-escalation study J Clin Oncol 2007 25 34 5410 5417 10.1200/JCO.2007.11.7960 18048823

[B5] Caldas-Lopes E Cerchietti L Ahn JH Clement CC Robles AI Rodina A Moulick K Taldone T Gozman A Guo Y Hsp90 inhibitor PU-H71, a multimodal inhibitor of malignancy, induces complete responses in triple-negative breast cancer models Proc Natl Acad Sci USA 2009 106 20 8368 8373 10.1073/pnas.0903392106 19416831 PMC2688867

[B6] Bao R Lai CJ Qu H Wang D Yin L Zifcak B Atoyan R Wang J Samson M Forrester J CUDC-305, a novel synthetic HSP90 inhibitor with unique pharmacologic properties for cancer therapy Clin Cancer Res 2009 15 12 4046 4057 10.1158/1078-0432.CCR-09-0152 19509149

[B7] Maloney A Workman P HSP90 as a new therapeutic target for cancer therapy: the story unfolds Expert Opin Biol Ther 2002 2 1 3 24 10.1517/14712598.2.1.3 11772336

[B8] Neckers L Heat shock protein 90: the cancer chaperone J Biosci 2007 32 3 517 530 10.1007/s12038-007-0051-y 17536171

[B9] Yano M Naito Z Tanaka S Asano G Expression and roles of heat shock proteins in human breast cancer Jpn J Cancer Res 1996 87 9 908 915 8878452 10.1111/j.1349-7006.1996.tb02119.x PMC5921196

[B10] Grbovic OM Basso AD Sawai A Ye Q Friedlander P Solit D Rosen N V600E B-Raf requires the Hsp90 chaperone for stability and is degraded in response to Hsp90 inhibitors Proc Natl Acad Sci USA 2006 103 1 57 62 10.1073/pnas.0609973103 16371460 PMC1325013

[B11] Shimamura T Lowell AM Engelman JA Shapiro GI Epidermal growth factor receptors harboring kinase domain mutations associate with the heat shock protein 90 chaperone and are destabilized following exposure to geldanamycins Cancer Res 2005 65 14 6401 6408 10.1158/0008-5472.CAN-05-0933 16024644

[B12] da Rocha Dias S Friedlos F Light Y Springer C Workman P Marais R Activated B-RAF is an Hsp90 client protein that is targeted by the anticancer drug 17-allylamino-17-demethoxygeldanamycin Cancer Res 2005 65 23 10686 10691 10.1158/0008-5472.CAN-05-2632 16322212

[B13] Blagosklonny MV Hsp-90-associated oncoproteins: multiple targets of geldanamycin and its analogs Leukemia 2002 16 4 455 462 10.1038/sj.leu.2402415 11960322

[B14] Kamal A Thao L Sensintaffar J Zhang L Boehm MF Fritz LC Burrows FJ A high-affinity conformation of Hsp90 confers tumour selectivity on Hsp90 inhibitors Nature 2003 425 6956 407 410 10.1038/nature01913 14508491

[B15] Mitsiades CS Mitsiades NS McMullan CJ Poulaki V Kung AL Davies FE Morgan G Akiyama M Shringarpure R Munshi NC Antimyeloma activity of heat shock protein-90 inhibition Blood 2006 107 3 1092 1100 10.1182/blood-2005-03-1158 16234364 PMC1895907

[B16] Senju M Sueoka N Sato A Iwanaga K Sakao Y Tomimitsu S Tominaga M Irie K Hayashi S Sueoka E Hsp90 inhibitors cause G2/M arrest associated with the reduction of Cdc25C and Cdc2 in lung cancer cell lines J Cancer Res Clin Oncol 2006 132 3 150 158 10.1007/s00432-005-0047-7 16283383 PMC12161051

[B17] Schwock J Pham NA Cao MP Hedley DW Efficacy of Hsp90 inhibition for induction of apoptosis and inhibition of growth in cervical carcinoma cells in vitro and in vivo Cancer Chemother Pharmacol 2008 61 4 669 681 10.1007/s00280-007-0522-8 17579866

[B18] Williams CR Tabios R Linehan WM Neckers L Intratumor injection of the Hsp90 inhibitor 17AAG decreases tumor growth and induces apoptosis in a prostate cancer xenograft model J Urol 2007 178 4 Pt 1 1528 1532 10.1016/j.juro.2007.05.120 17707057

[B19] Solit DB Osman I Polsky D Panageas KS Daud A Goydos JS Teitcher J Wolchok JD Germino FJ Krown SE Phase II trial of 17-allylamino-17-demethoxygeldanamycin in patients with metastatic melanoma Clin Cancer Res 2008 14 24 8302 8307 10.1158/1078-0432.CCR-08-1002 19088048 PMC2629404

[B20] Lewin DA Weiner MP Molecular biomarkers in drug development Drug Discov Today 2004 9 22 976 983 10.1016/S1359-6446(04)03272-6 15539141

[B21] Rolan P Atkinson AJ Jr Lesko LJ Use of biomarkers from drug discovery through clinical practice: report of the Ninth European Federation of Pharmaceutical Sciences Conference on Optimizing Drug Development Clin Pharmacol Ther 2003 73 4 284 291 10.1016/S0009-9236(02)17625-9 12709718

[B22] Dancey JE Dobbin KK Groshen S Jessup JM Hruszkewycz AH Koehler M Parchment R Ratain MJ Shankar LK Stadler WM Guidelines for the development and incorporation of biomarker studies in early clinical trials of novel agents Clin Cancer Res 16 6 1745 1755 10.1158/1078-0432.CCR-09-2167 20215558

[B23] Kulasingam V Diamandis EP Strategies for discovering novel cancer biomarkers through utilization of emerging technologies Nat Clin Pract Oncol 2008 5 10 588 599 10.1038/ncponc1187 18695711

[B24] Clarke PA Hostein I Banerji U Stefano FD Maloney A Walton M Judson I Workman P Gene expression profiling of human colon cancer cells following inhibition of signal transduction by 17-allylamino-17-demethoxygeldanamycin, an inhibitor of the hsp90 molecular chaperone Oncogene 2000 19 36 4125 4133 10.1038/sj.onc.1203753 10962573

[B25] Maloney A Clarke PA Naaby-Hansen S Stein R Koopman JO Akpan A Yang A Zvelebil M Cramer R Stimson L Gene and protein expression profiling of human ovarian cancer cells treated with the heat shock protein 90 inhibitor 17-allylamino-17-demethoxygeldanamycin Cancer Res 2007 67 7 3239 3253 10.1158/0008-5472.CAN-06-2968 17409432

[B26] Song D Chaerkady R Tan AC Garcia-Garcia E Nalli A Suarez-Gauthier A Lopez-Rios F Zhang XF Solomon A Tong J Antitumor activity and molecular effects of the novel heat shock protein 90 inhibitor, IPI-504, in pancreatic cancer Mol Cancer Ther 2008 7 10 3275 3284 10.1158/1535-7163.MCT-08-0508 18852131

[B27] Banerji U O'Donnell A Scurr M Pacey S Stapleton S Asad Y Simmons L Maloney A Raynaud F Campbell M Phase I pharmacokinetic and pharmacodynamic study of 17-allylamino, 17-demethoxygeldanamycin in patients with advanced malignancies J Clin Oncol 2005 23 18 4152 4161 10.1200/JCO.2005.00.612 15961763

[B28] Benjamini Y Drai D Elmer G Kafkafi N Golani I Controlling the false discovery rate in behavior genetics research Behav Brain Res 2001 125 1-2 279 284 10.1016/S0166-4328(01)00297-2 11682119

[B29] Glez-Pena D Gomez-Lopez G Pisano DG Fdez-Riverola F WhichGenes: a web-based tool for gathering, building, storing and exporting gene sets with application in gene set enrichment analysis Nucleic Acids Res 2009 37 Web Server W329 334 10.1093/nar/gkp263 19406925 PMC2703947

[B30] Hu Z Fan C Livasy C He X Oh DS Ewend MG Carey LA Subramanian S West R Ikpatt F A compact VEGF signature associated with distant metastases and poor outcomes BMC Med 2009 7 9 10.1186/1741-7015-7-9 19291283 PMC2671523

[B31] Zajac M Moneo MV Carnero A Benitez J Martinez-Delgado B Mitotic catastrophe cell death induced by heat shock protein 90 inhibitor in BRCA1-deficient breast cancer cell lines Mol Cancer Ther 2008 7 8 2358 2366 10.1158/1535-7163.MCT-08-0327 18723483

[B32] Amundson SA Bittner M Chen Y Trent J Meltzer P Fornace AJ Jr Fluorescent cDNA microarray hybridization reveals complexity and heterogeneity of cellular genotoxic stress responses Oncogene 1999 18 24 3666 3672 10.1038/sj.onc.1202676 10380890

[B33] Fambrough D McClure K Kazlauskas A Lander ES Diverse signaling pathways activated by growth factor receptors induce broadly overlapping, rather than independent, sets of genes Cell 1999 97 6 727 741 10.1016/S0092-8674(00)80785-0 10380925

[B34] Kelland LR Sharp SY Rogers PM Myers TG Workman P DT-Diaphorase expression and tumor cell sensitivity to 17-allylamino, 17-demethoxygeldanamycin, an inhibitor of heat shock protein 90 J Natl Cancer Inst 1999 91 22 1940 1949 10.1093/jnci/91.22.1940 10564678

[B35] Gaspar N Sharp SY Pacey S Jones C Walton M Vassal G Eccles S Pearson A Workman P Acquired resistance to 17-allylamino-17-demethoxygeldanamycin (17-AAG, tanespimycin) in glioblastoma cells Cancer Res 2009 69 5 1966 1975 10.1158/0008-5472.CAN-08-3131 19244114 PMC2652695

[B36] Neve RM Chin K Fridlyand J Yeh J Baehner FL Fevr T Clark L Bayani N Coppe JP Tong F A collection of breast cancer cell lines for the study of functionally distinct cancer subtypes Cancer Cell 2006 10 6 515 527 10.1016/j.ccr.2006.10.008 17157791 PMC2730521

[B37] Han Y Shen H Carr BI Wipf P Lazo JS Pan SS NAD(P)H:quinone oxidoreductase-1-dependent and -independent cytotoxicity of potent quinone Cdc25 phosphatase inhibitors J Pharmacol Exp Ther 2004 309 1 64 70 10.1124/jpet.103.059477 14718602

[B38] Powers MV Clarke PA Workman P Dual targeting of HSC70 and HSP72 inhibits HSP90 function and induces tumor-specific apoptosis Cancer Cell 2008 14 3 250 262 10.1016/j.ccr.2008.08.002 18772114

[B39] Steiner T Junker U Henzgen B Nuske K Durum SK Schubert J Interferon-alpha suppresses the antiapoptotic effect of NF-kB and sensitizes renal cell carcinoma cells in vitro to chemotherapeutic drugs Eur Urol 2001 39 4 478 483 10.1159/000052489 11306890

[B40] Lind DS Hochwald SN Malaty J Rekkas S Hebig P Mishra G Moldawer LL Copeland EM Mackay S Nuclear factor-kappa B is upregulated in colorectal cancer Surgery 2001 130 2 363 369 10.1067/msy.2001.116672 11490372

[B41] Hernandez-Vargas H Rodriguez-Pinilla SM Julian-Tendero M Sanchez-Rovira P Cuevas C Anton A Rios MJ Palacios J Moreno-Bueno G Gene expression profiling of breast cancer cells in response to gemcitabine: NF-kappaB pathway activation as a potential mechanism of resistance Breast Cancer Res Treat 2007 102 2 157 172 10.1007/s10549-006-9322-9 17039268

[B42] Biswas DK Shi Q Baily S Strickland I Ghosh S Pardee AB Iglehart JD NF-kappa B activation in human breast cancer specimens and its role in cell proliferation and apoptosis Proc Natl Acad Sci USA 2004 101 27 10137 10142 10.1073/pnas.0403621101 15220474 PMC454178

[B43] Dong G Chen Z Kato T Van Waes C The host environment promotes the constitutive activation of nuclear factor-kappaB and proinflammatory cytokine expression during metastatic tumor progression of murine squamous cell carcinoma Cancer Res 1999 59 14 3495 3504 10416616

[B44] Arlt A Grobe O Sieke A Kruse ML Folsch UR Schmidt WE Schafer H Expression of the NF-kappa B target gene IEX-1 (p22/PRG1) does not prevent cell death but instead triggers apoptosis in Hela cells Oncogene 2001 20 1 69 76 10.1038/sj.onc.1204061 11244505

[B45] Arlt A Gehrz A Muerkoster S Vorndamm J Kruse ML Folsch UR Schafer H Role of NF-kappaB and Akt/PI3K in the resistance of pancreatic carcinoma cell lines against gemcitabine-induced cell death Oncogene 2003 22 21 3243 3251 10.1038/sj.onc.1206390 12761494

[B46] Biswas DK Martin KJ McAlister C Cruz AP Graner E Dai SC Pardee AB Apoptosis caused by chemotherapeutic inhibition of nuclear factor-kappaB activation Cancer Res 2003 63 2 290 295 12543776

