# Supplementary material for: Molecular signature of response and potential pathways related to resistance to the HSP90 inhibitor, 17AAG, in breast cancer
Source: BMC Med Genomics. 2010 Oct 4;3:44. doi: 10.1186/1755-8794-3-44 (PMC2959047; doi:10.1186/1755-8794-3-44)
Supplement: Additional file 1 — Supplemental tables and figures. Supplemental Table S1: Oligonucleotides used for quantitative RT-PCR to validate expression of the selected from microarray study. Supplemental Table S2: List of the antibodies used for western blot. Supplemental Table S3: Top-ten pathways more associated to resistance to 17AAG. Supplemental Figure S1: Unsupervised clustering showed that samples mostly grouped by cell line. Some samples have technical replicates (marked with a star). Supplemental Figure S2: QT-PCR measurements of selected genes from the signature, confirmed the down regulation of five genes from the molecular signature (CYCLIN D1, JUNB, TMEM129, PLK3, NFKBIA) and up-regulation of UBE2C after treatment. [file 1755-8794-3-44-S1.DOC]

**ADDITIONAL FILE 1.**

**Supplemental Table S1.** Oligonucleotides used for quantitative RT-PCR to validate expression of the selected from microarray study.

|  |  |
| --- | --- |
| Gene name | Primer pair sequence |
| CCND1 | Left Primer gaagatcgtcgccacctg |
|  | Right Primer gacctcctcctcgcacttct |
| JUNB | Left Primer atacacagctacgggatacgg |
|  | Right Primer gctcggtttcaggagtttgt |
| NFKBIA | Left Primer gtcaaggagctgcaggagat |
|  | Right Primer gatggccaagtgcaggaa |
| PLK3 | Left Primer gaaggtgggggattttgg |
|  | Right Primer gggtgccacagatggtct |
| TMEM129 | Left Primer cagcaccaaccctgctgt |
|  | Right Primer ggtcttcaccagcttcacg |
| UBE2C | Left Primer ctggaaaaaccccacagc |
|  | Right Primer aaaagacgacacaaggacagg |

|  |  |
| --- | --- |
| Gene name | Primer pair sequence |
| RGS2 | Left Primer caaacagcaagctttcatcaag |
|  | Right Primer cctgaatgcagcaagacca |
| CHORDC1 | Left Primer gccagtagaagcaataaaaagacc |
|  | Right Primer tgatgacagtttaagtttatcaagtgc |
| DEDD2 | Left Primer accccagcagcagtcaga |
|  | Right Primer ctcgcagtactctgctcgaa |
| DNAJA1 | Left Primer ccatggtgaaggagaccaa |
|  | Right Primer catgaaaaggtcttctcctcgt |
| DNAJB12 | Left Primer tccacccagacaagaaccac |
|  | Right Primer cgcatatgctgtgccaat |
| HSP90AA1 | Left Primer gatgatgagcagtacgcttgg |
|  | Right Primer cttttgttccacgacccatag |
| HSPA1L | Left Primer agggagcccccacctact |
|  | Right Primer gattcccttggcagtagcc |
| HSPA4L | Left Primer cctcttttgaagatggaagtgg |
|  | Right Primer tggttccttcttgtggaaagtaa |
| HSPA8 | Left Primer tttttgtggcttccttcgtt |
|  | Right Primer tcccttggacatggttgc |
| MICB | Left Primer ctgagaaggtggcgacgta |
|  | Right Primer cgaagactgtggggctca |
|  |  |

**Supplemental Table S2**: Description of antibodies used for western blotting

|  |  |  |  |  |
| --- | --- | --- | --- | --- |
| **Name** | **Clone** | **Host** | **Dilution** | **Provider** |
| Hsp70 | W27 | Mouse | 1/500 | Santa Cruz Biotechnology |
| Hspa2 | ab55290 | Mouse | 1/500 | Abcam |
| Hspa1L | 7HG | Mouse | 1/200 | AbNova |
| Hsc70 | N27F3-4 | Mouse | 1/1000 | Stressgen |
| Hsp72 | C92F3A-5 | Mouse | 1/1000 | Stressgen |
| GAPDH | 220/EG3 | Mouse | 1/50 | Monoclonal Antibody Unit, CNIO |
|  |  |  |  |  |

**Supplemental Table S3. List of pathways more associated to resistance to 17AAG after GSEA.**

| **NAME** | **Description ( from Molecular Signatures database)** | **FDR q-val** |
| --- | --- | --- |
| CDMAC_PATHWAY | Cadmium 2+ promotes cell proliferation in cultured macrophages by entering the cell via calcium channels and activating the MAP kinase pathway. | 0.157 |
| NFKB_PATHWAY | Inactive nuclear factor kB (NF-kB) is inhibited by the IkB family in the cytoplasm; active NF-kB is localized in the nucleus and regulates transcription of a variety of genes | 0.204 |
| ATM_PATHWAY | The tumor-suppressing protein kinase ATM responds to radiation-induced DNA damage by blocking cell-cycle progression and activating DNA repair | 0.245 |
| CHREBP_PATHWAY | Carbohydrate responsive element binding protein (chREBP) is a transcription factor inhibited by cAMP and activated by high carbohydrate levels | 0.250 |
| NTHI_PATHWAY | Hemophilus influenzae infections activate NF-kB via several pathways, inducing the inflammatory response. | 0.257 |
| INFLAM_PATHWAY | Interleukins and TNF serve as signals to coordinate the inflammatory response, in which macrophages recruit and activate neutrophils, fibroblasts, and T cells. | 0.329 |
| WNT_PATHWAY | The Wnt glycoprotein binds to membrane-bound receptors such as Frizzled to activate a number of signaling pathways, including that of beta-catenin | 0.397 |
| CCR5_PATHWAY | CCR5 is a G-protein coupled receptor expressed in macrophages that recognizes chemokine ligands and is targeted by the HIV envelope protein GP120 | 0.440 |
| CARDIACEGF_PATHWAY | Cardiac hypertrophy, a response to high blood pressure, is stimulated by GPCR ligands such as angiotensin II that activate the EGF pathway | 0.468 |
| VIP_PATHWAY | Apoptosis of activated T cells is inhibited by vasoactive intestinal peptide (VIP) and its relative PACAP | 0.480 |

**Supplemental Figure S1. Unsupervised cluster with more than 20000 genes of all control and treated, 24h and 48h, samples.** Samples mostly grouped by cell line. Some samples have technical replicates (marked with a star).

**Supplemental Figure S2.** **Validation of the molecular signature of response to 17-AAG**. QT-PCR measurements of selected genes from the signature, confirmed the down regulation of five genes from the molecular signature (*CYCLIN D1, JUNB, TMEM129, PLK3, NFKBIA*) and up-regulation of *UBE2C* after treatment.
